# A case report of anorectal malignant melanoma in the transitional zone

**DOI:** 10.1016/j.ijscr.2020.09.091

**Published:** 2020-09-17

**Authors:** Minh Thao Nguyen, Van Mao Nguyen, Van Huy Tran, Anh Vu Pham

**Affiliations:** a Digestive Surgery Department, Hue University of Medicine and Pharmacy Hospital, Hue University of Medicine and Pharmacy, Hue University, 49126, Hue City, Viet Nam; b Pathology Department, Hue University of Medicine and Pharmacy Hospital, Hue University of Medicine and Pharmacy, Hue University, 49126, Hue City, Viet Nam; c Department of Internal Medicine, Hue University of Medicine and Pharmacy, Hue University, 49126, Hue City, Viet Nam

**Keywords:** Anorectal melanoma, Transitional zone, Case report

## Abstract

**Introduction:**

Anorectal malignant melanoma is an uncommon and highly malignant disease with a greater incidence in females. Many patients were misdiagnosed as hemorrhoids, benign polyps, and rectal cancer. They were often diagnosed in an advanced stage. Wide local excision and abdominoperineal resection are the main treatments of rectal melanoma.

**Presentation of case:**

A case report is a 77-year-old man who has blood in the stool for 4 months without clinical examination. He admitted to the emergency room with sudden syndromes that related to bowel perforation. Rectal examination detected a large anorectal polyp. Computer tomography showed free air and fluid in the peritoneal cavity. He was received laparoscopic surgery and found the fishbone penetrated the sigmoid colon without polyp resection. The polyp was treated by local excision a few days later. The histology examination was a primary malignant melanoma. Due to the pigmented lesion that remained from the resected polyp’s root, the abdominoperineal resection was performed as a radical treatment.

**Discussion:**

Diagnosis of anorectal malignant melanoma is difficult because of atypical signs, that are confused with bleeding hemorrhoids especially an amelanotic melanoma. Treatment is controversial, including surgery, radiotherapy, chemotherapy, and target therapy. A present case is an option in radical surgery.

**Conclusion:**

Anorectal melanoma is a rare disease with poor results and prognosis. A lack of large-data leads to a missing evidence-based guideline in this disease. Early-staging diagnosis and surgical treatment help patients improve their overall survival.

## Highlights

• Anorectal melanoma is a rare disease with poor results and prognosis.
• Patients are misdiagnosed as hemorrhoids, benign polyps, and even rectal cancer.
• Early-staging diagnosis and surgical treatment are important to improve overall survival.

## 1 Introduction

Anorectal malignant melanoma (ARM) is an extremely rare and highly malignant tumor with a 5-year survival rate being 10% [1,2] About 1% of malignant melanomas are anorectal cancer and 1% of rectal cancer is malignant melanoma, that is the third most common primary of melanoma after skin and retina [3,4].

Due to the location of the tumor and the deficiency of symptoms, patients are often diagnosed at an advanced stage. Two-thirds of patients were diagnosed incorrectly as hemorrhoids, adenocarcinoma polyps, and rectal cancer [1,4]. ARM patients are more common in females, the adults older than 60 years [1,3].

Treatments of rectal melanoma are controversial. Surgery, wide local excision (WLE), abdominoperineal resection (APR), and endoscopic mucosal resection (EMR) have been widely adopted as the primary treatment [1,3,4]. Chemotherapy, radiation therapy, immunotherapy provide uncertain results [1,5]. Recent advances target therapy and anti-angiogenetic therapy have also been fully elucidated [1,4].

The lack of medical evidence of etiology, pathogenesis, and genetics of ARM makes for difficulties in an accurate diagnosis, treatment, and prognosis [1].

In this article, we report a case of melanoma in the transitional zone with a complicated history. This case report followed SCARE guidelines [21].

## 2 Case presentation

**Fig. 1:**
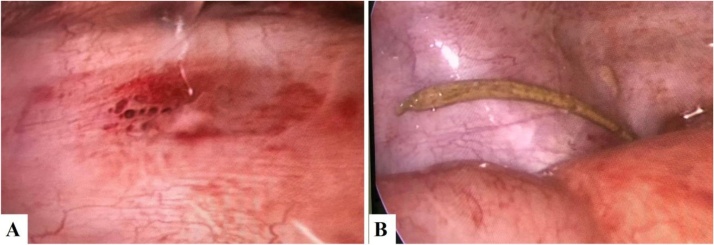
A. The lesion in the sigmoid colon. B. An image with a fishbone penetrates to the peritoneal cavity.

A 77-year-old man has a history with blood in the stool for 4 months without clinical examination. He presented with a sudden onset of severe, sharp abdominal pain. He described generalized pain and fever 10 h before hospitalization. There was no nausea, vomiting, and diarrhea. Physical examination revealed tenderness at the hypogastric region, and rectal examination detected a large anorectal polyp. The white blood cell count was 161 G/l with 92% neutrophil. An erect abdominal X-ray revealed a peritoneal free air underneath the left diaphragm. Abdominal ultrasound (US) exposed minimal free fluid in the pelvic region. The whole abdominal CT showed free air and fluid in the peritoneal cavity. Therefore, laparoscopic surgery was indicated to resolve that. The fishbone that was found beside the penetrated sigmoid wall would be removed later. He was sutured the perforated hole in the sigmoid colon and performed ileostomy (Fig. 1).

**Fig. 2:**
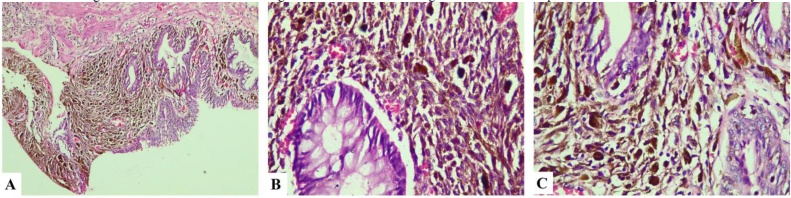
Histological examination of polyp showing the atypical melanocytic cell with pigmented debris in cytoplasm infiltrating the gland. A: H.E x 100; B and C: H.E x 400.

**Fig. 3:**
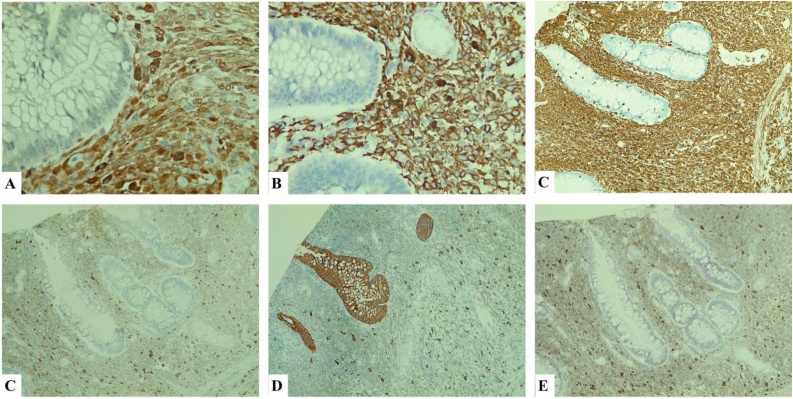
Immunohistochemistry A. S100 × 400 pos; B. HMB45 × 400 pos, C. Vimentin x 100 pos, D. CD68 × 100 neg, E. AE1/3 × 100 neg, F. P53 × 100 + sporadic.

The patient’s symptoms were improved after 5 days. Due to the age of patient and to his history of rectal bleeding, a colorectal endoscopy was indicated and revealed the large pigmented polyp in the transitional zone of the anorectal area and some small polyps in the colon. These small polyps were removed at the moment by colonoscopy. The large pigmented polyp was removed subsequently by local excision. Histopathology showed features of malignant melanoma (Fig. 2). And Immunohistochemical results determined the diagnosis of colorectal mucosa melanoma with Protein S-100, HMB45, vimentin-positive; AE1/3, CD68, and P53 negative (Fig. 3).

**Fig. 4:**
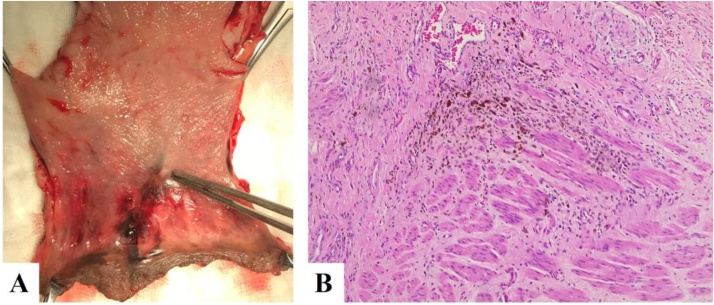
A. Postoperative piece. The resected polyp’s root with pigmented lesion spreading out the dentate line (surgical specimen). B. H.E x 100. Histopathology showed the pigmented atypical melanocytic cell infiltrating into the muscle with 7 mm of thickness.

He was supported and improved his health with oral and vein nutrition therapy. His overall condition was checked, and the malignancy-risk was calculated with chest, abdominal, pelvis CT scan. These examinations couldn’t find metastasis lesions. After the total body skin, ocular-retina, and nasopharynx examination, two pigmented skin lesions were detected and removed later. Histopathological results were benign pigmented tumors (Fig. 4).

Two weeks after the local excision of the polyp, a 2nd colorectal endoscopy was done and showed a pigmented image spreading out from the resected polyp’s root that had been removed before. A pelvis MRI and anorectal endoscopic ultrasound (EUS) evaluated this remain lesion was limited in mucosa and submucosa, his anal canal staging was cT1N0. The patient was performed radical resection with abdominoperineal excision (APR) and total mesorectal excision (TME) by laparoscopic surgery + closure of loop ileostomy.

## 3 Discussion

### 3.1 Origin and diagnosis

The hindgut includes from the distal third of the transverse colon to the upper part of the anal canal; the distal part of the anal canal initiated from ectoderm. The dentate line, the line which divides the upper and the lower anal canal is defined by the junction between the endodermal and ectodermal. At this line, the columnar epithelium in the upper anal canal (transitional zone) and colorectal region changes to the stratified epithelium in the lower (squamous zone). Melanocytes are located in the squamous zone, in the transitional zone, and never in the colorectal zone. They originate from the embryonic ectoderm cells called neural crest cells that migrate to the distal ileum through the umbilical-mesenteric canal. Therefore, they were presented in the squamous, transitional, and colorectal zone. The ileum that originates from this canal should be the most rate in primary malignant melanoma of the small bowel. Otherwise, ARM originates from melanocytes mostly near the area of the dentate line and penetrates the distal rectal mucosa [1,6].

Diagnosis of ARM is difficult because of atypical signs, that are confused with bleeding hemorrhoids especially an amelanotic melanoma [7]. Sentinel lymph node (SLN) mapping and biopsy in ARM have not been recommended for this disease [2,[8], [9], [10]]. However, SLN detection may be useful for the staging and treatment of ARM in patients with a middle thickness tumor [7].

### 3.2 Histology

Histology and immunohistochemistry are important for diagnosis. Histological examination describes the pigmented lesions is based on the presence and location of atypical melanocytes. Immunohistochemical studies are mostly positive for protein S-100, melanoma antigen HMB-45, and vimentin, whereas positive staining for carcinoembryonic antigen, cytokeratin, epithelial membrane antigen is suggestive of adenocarcinoma [11,9].

### 3.3 Staging and prognosis

The staging of ARM differs from the cutaneous melanoma. Most researches used the clinical anorectal staging with stage I: local disease, stage II: a local disease with positive regional lymph nodes, and stage III: distant metastasis. Patients with stage I, II were not of the same grade but they also received WLE or APR in this researches. Besides, due to the limited number of cases that are studied and the different selections of surgery in each stage as to whether or not to remove sentinel lymph nodes, it is difficult to prove their advantages (Table 1). However, the other staging based on TNM classification in the 8th American Joint Committee on Cancer (AJCC), is divided into two regions: rectum and anus. The rectal TNM staging is based on the depth of tumor (T) invasion into or beyond the wall of the rectum, the number of regional lymph nodes (N), and the presence or absence of distant metastasis (M). The anal region has three different histologic types: glandular, transitional, and squamous cells. For staging purposes, tumors should be classified as rectal cancers if they are located above 2 cm from the dentate line and as anal canal cancers if they are located below. The anal TNM differs from rectal TNM in the greatest dimension of primary tumors (T), lymph-nodal involvement in perirectal, unilateral internal iliac, and/or inguinal region. The TNM classification is used for both clinical and pathologic staging. Stage I and II: localized tumors, stage III: regional lymph node metastasis, and stage IV: distant disease. In the seventh edition, further substaging of TNM classification has been accomplished, based on survival and relapse data that was analyzed better than prior general stagings [[12], [13], [14]].

**Table 1:** Case reports of anorectal melanoma.

Author/Nation	n	Age m,(r)	Stage	Treatment	Recurence	Survival(Mo)
Hick 2014/USA	18	64 (45–74)	I: 10	WLE:11	11/13	15,5
			II: 5	APR: 7		
			III: 3			
Doods 2018/Australia	43	61 (28–89)	I: 1	LE:15	6 local	9
			II: 13	APR: 20	10 regional lymph nodes	
			III: 21	Unknown: 3	15 distance	
			IV 6	Biopsy only: 4		
Roumen 1996/Netherlands	63	66 (29–89)	I: 35	WLE:16	N.A	28(grade I)
						16(grade II)
						4(grade III)
			II: 7	APR: 18		
			III: 21			
Ramakrishnan 2008/India	63	53 (32–79)	I: 11	WLE:8	5(WLE)	8(WLE)
			II: 16	WLE + RT: 34	3(WLE + RT)	28(WLE + RT)
			III: 36	APR: 3	3(APR)	5(APR)
				RT: 3		0(RT)

m: mean, r: range, WLE: Wide local excision, LE: Local excision, APR: Abdominoperineal resection, RT: Adjuvant radiation.

The thickness of the tumor is associated with prognosis, Some authors believed patients that have longer survival related to the thickness of the tumor less than 4 mm. Thus, the thickness of 4 mm or more accords with advanced disease with shortened survival [2,8,15].

### 3.4 Treatment

Management of ARM is controversial, including surgery, radiotherapy, chemotherapy, and target therapy. Many reports had approved surgery as a primary choice to control melanotic neoplasms, but these researches show uncertain results because of the insufficiency of the randomized trial. Mucosal resection (EMR) is performed by a few authors as the second choice with long-term survival achieved in several cases (>6years) [16]. Although conventional APR is considered the main option for local region treatment with long-term survival, there is no significant benefit versus WLE [1,4,16]. In recent years, WLE is chosen widely with lower mortality but the outcome is not impacted. However, success with R0 resection in WLE was important in some reports showed longer survival than APR although the outcome based on the early staging diseases that were elected in WLE [3,17]. Some researches showed the safety resection margin in WLE is 1–2 cm surrounding the tumor. In tumor thickness less than 1 mm, the local margin with sphincter-saving excision is 1 cm, if the tumor thickness from 1–4 mm, the adequate margin is 2 cm, if the tumor thickness above 4 mm, a WLE doesn’t seem to be the sufficient management for ARM [16,18,19]. So, frozen biopsy during operation is a routine to achieve a sufficient negative margin. Besides, adjuvant therapies were effective for ARM. Ramakrishnan et al. showed the outcome of patients received WLE + RT is better than other groups (Table 1) [9]. In advanced diseases, adjuvant therapy had demonstrated a significant benefit of α-interferon upon relapse-free survival and overall survival [1,20]. It is a fact that target therapy has an increasingly significant benefit to survival, but the outcomes are still unconvincing, and that is the trend for future perspective [1,4].

## 4 Conclusions

Anorectal melanoma is a rare disease with poor outcome and prognosis. The lack of high-volume data researches in the anorectal melanoma field shows the missing guideline for this disease. Early-staging diagnosis and surgical management help patients with anorectal melanoma diseases improve their overall survival.

## Declaration of Competing Interest

None declared. The authors have no financial, consultative, institutional, and other relationships that might lead to bias or conflict of interest.

## Funding

This research did not receive any specific grant from funding agencies in the public, commercial, or not-for-profit sectors.

## Ethical approval

There is no ethical approval was obtained as it’s a case report but a written consent was taken from the patient.

## Consent

Written informed consent was obtained from the patient for publication of this case report and accompanying images. A copy of the written consent is available for review by the Editor-in-Chief of this journal on request.

## Author contribution

Minh Thao Nguyen: Concept and design of study, data collection, data interpretation and analysis, drafting, writing the paper, revision, approval of final manuscript.

Van Mao Nguyen: Data collection, approval of final manuscript.

Van Huy Tran: Data collection, approval of final manuscript.

Anh Vu Pham: Data interpretation and analysis, drafting, revision, approval of final manuscript.

## Registration of research studies

Our paper is a case report, no registration was done for it.

## Guarantor

Minh Thao Nguyen: nmthao@huemed-univ.edu.vn.

Anh Vu Pham: pavu@huemed-univ.edu.vn.

## Provenance and peer review

Not commissioned, externally peer-reviewed.
